# Chest Wall Synovial Sarcoma: A Unique Encounter at the Breast Base

**DOI:** 10.7759/cureus.63499

**Published:** 2024-06-30

**Authors:** Rana Bilal Idrees, Mariam Malik, Farwa Malik, Bareera Rehman, Taimoor Sarwar, Ahmed Mustansar, Muhammad Hamid Chaudhary

**Affiliations:** 1 Radiology, Institute of Nuclear Medicine and Oncology Lahore (INMOL) Cancer Hospital Lahore, Lahore, PAK; 2 Radiology, Atomic Energy Cancer Hospital, Nuclear Medicine, Oncology and Radiotherapy Institute (NORI), Islamabad, PAK; 3 Cardiac Surgery, Chaudhry Pervaiz Elahi Institute of Cardiology, Multan, PAK

**Keywords:** chest wall sarcoma, cutaneous oncology, chest wall tumour, general radiology, sarcoma soft tissue

## Abstract

Synovial sarcomas most commonly arise in the para-articular locations of the extremities, such as the upper limbs, thigh, knee, ankle, and foot. Thoracic synovial sarcomas are a rare entity that can arise in the chest wall, pleura, lung, heart, or mediastinum. We present a case of a 23-year-old female with a complaint of swelling of the left breast. Examination demonstrated an enlarged left breast and a hard-fixed swelling without overlying skin changes or nipple retraction. Ultrasound showed a well-defined, solid-appearing lesion deep in the left breast parenchyma, which was adherent to the underlying left chest wall musculature and seemed to be displacing the breast parenchyma anteriorly. Contrast-enhanced computed tomography (CECT) and magnetic resonance imaging (MRI) confirmed the lesion centered at the left pectoralis major and minor muscles, confirming the chest wall's origin. Histopathology findings favored monophasic synovial sarcoma.

## Introduction

Synovial sarcomas are the most common non-rhabdomyosarcomatous soft tissue sarcomas in children and young adults [1] and have an equal gender preference [2]. However, the name is a misnomer, as studies have suggested a mesenchymal origin for these tumors [3]. They most commonly arise in para-articular locations of the extremities, such as the upper limbs, thigh, knee, ankle, and foot.

Thoracic synovial sarcoma is a rare entity that can arise in the chest wall, pleura, lung, heart, or mediastinum [4,5]. Data regarding the exact incidence in the chest wall is insufficient, as only a few cases have been reported in the literature, and there is often a lack of follow-up [3]. The disease is usually present for approximately two years before the onset of symptoms and is typically slow-growing [3]. The symptoms occur due to the invasion of the chest wall by the tumor or by pressure effects on the underlying structures.

## Case presentation

A 23-year-old female patient presented to surgical OPD with a complaint of swelling of the left breast. Examination demonstrated an enlarged left breast and a hard-fixed palpable mass. No overlying skin changes or nipple retractions were evident. She was referred to radiology for an ultrasound of the left breast.

**Figure 1:**
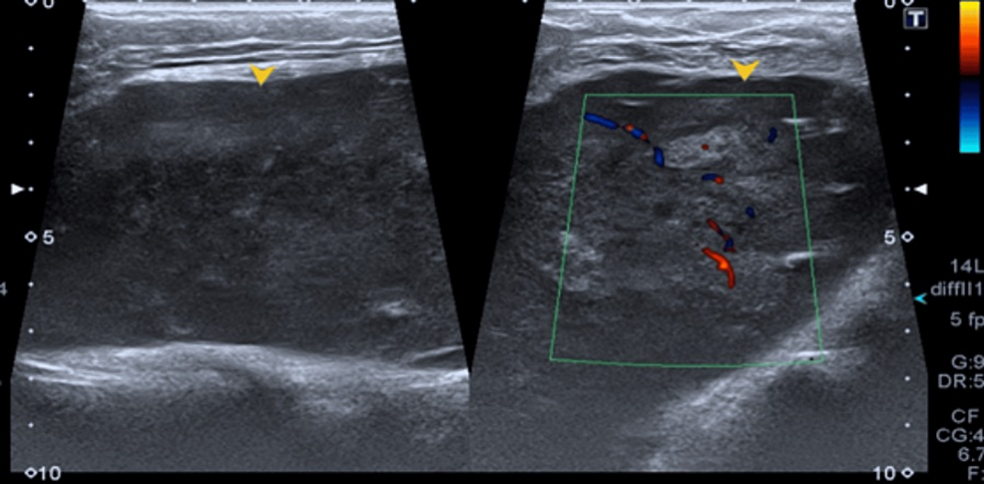
A well-defined heterogeneous hypoechoic lesion in left chest wall (as annotated by yellow arrowhead). The image on right shows internal vascularity appearing as linear intralesional red and blue areas.

Ultrasound showed a well-defined, solid-appearing lesion (Figure 1) with lobulated margins and internal cystic/necrotic areas deep in the left breast parenchyma. The lesion appeared adherent to the underlying left chest wall musculature and seemed to be displacing the breast parenchyma anteriorly.

**Figure 2:**
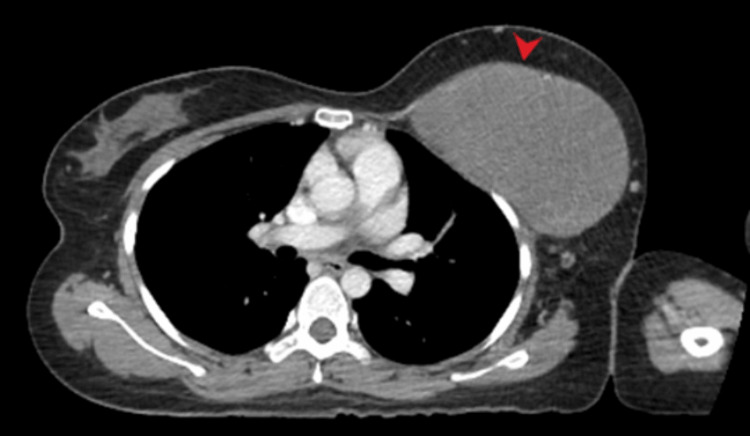
Axial post contrast CT chest at the level of upper chest shows a well-circumscribed soft tissue density lesion inseparable from underlying chest wall muscles (annotated by red arrow head).

**Figure 3:**
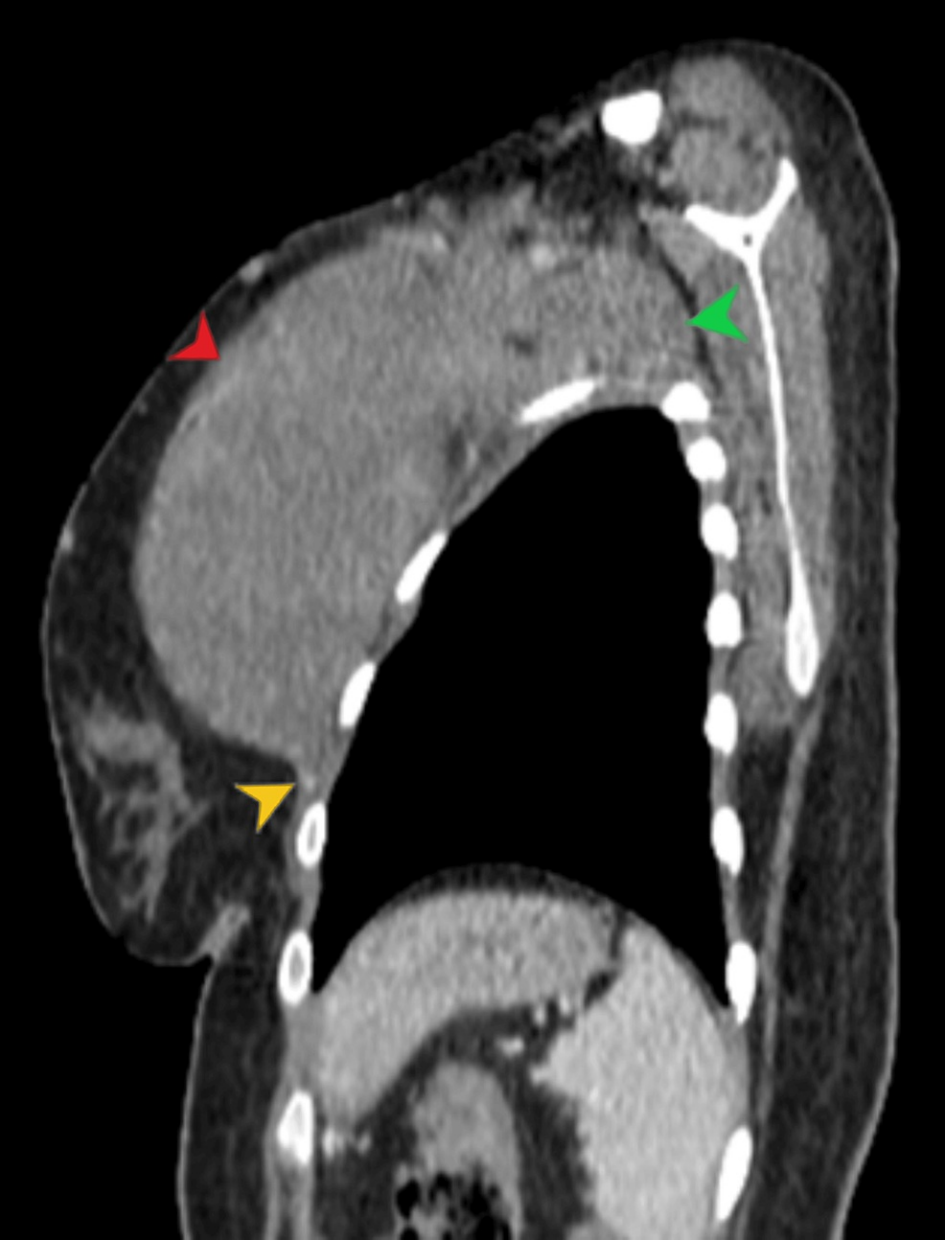
Sagittal post contrast CT chest demonstrates a well-circumscribed soft tissue density lesion annotated by red arrow head. The lesion is inseparable from underlying chest wall muscles (yellow arrow head). There is extension into the left axilla (green arrow head).

A contrast-enhanced computed tomography (CECT) correlation was recommended, which was subsequently done and showed an 8 cm × 12 cm × 14 cm large lesion centered at the left pectoralis major and minor muscles, confirming the chest wall origin (as shown in Figures 2-3). Breast parenchyma remained unremarkable. The mass extended into the left axilla; however, no ipsilateral axillary lymphadenopathy was evident. No pulmonary abnormality or pleural effusion was seen. No metastatic disease was found in the CECT abdomen and pelvis.

**Figure 4:**
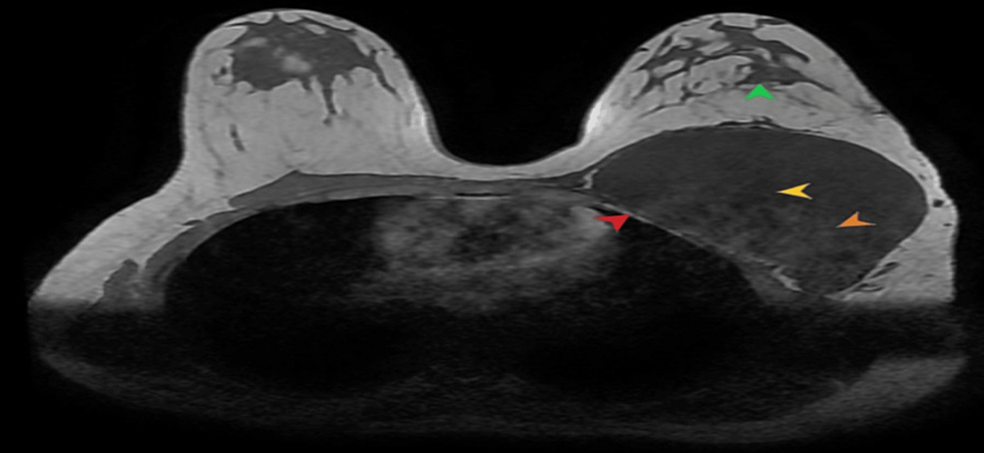
Non-contrast T1 axial MR image shows the lesion attached to left chest wall (annotated by red arrow head). Intralesional T1 low signal areas correspond to necrosis (yellow arrow head), while T1 hyperintense component correlates with hemorrhage (orange arrow head). The left breast parenchyma is displaced anteriorly (green arrow head).

**Figure 5:**
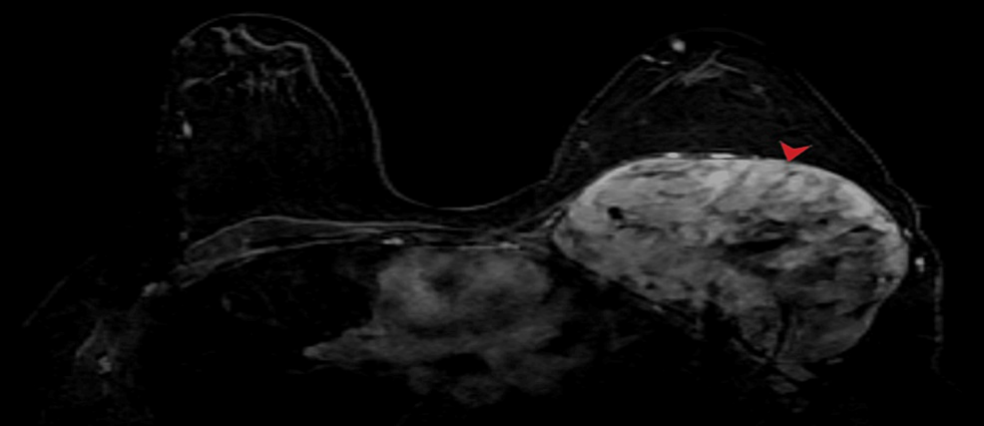
Post-contrast fat suppressed T1 axial MR image shows a heterogeneous post-contrast enhancement pattern due to presence of intra-tumoral necrotic changes that remain unenhanced.

Magnetic resonance imaging (MRI) of the chest wall was also done that confirmed similar findings with intralesional areas of hemorrhage that returned T1 hyperintense signals (Figure 4). There were intralesional areas of necrosis as well that appeared T1 hypointense and T2 hyperintense. Post contrast images showed heterogeneous enhancement (Figure 5).

Differential diagnoses of benign peripheral nerve sheath tumor, solitary fibrous tumor, fibrosarcoma, and synovial sarcoma were given, and histopathological correlation was advised for exact characterization. Hence, an ultrasound-guided core biopsy revealed tissue fragments comprising a spindle cell neoplasm.

**Figure 6:**
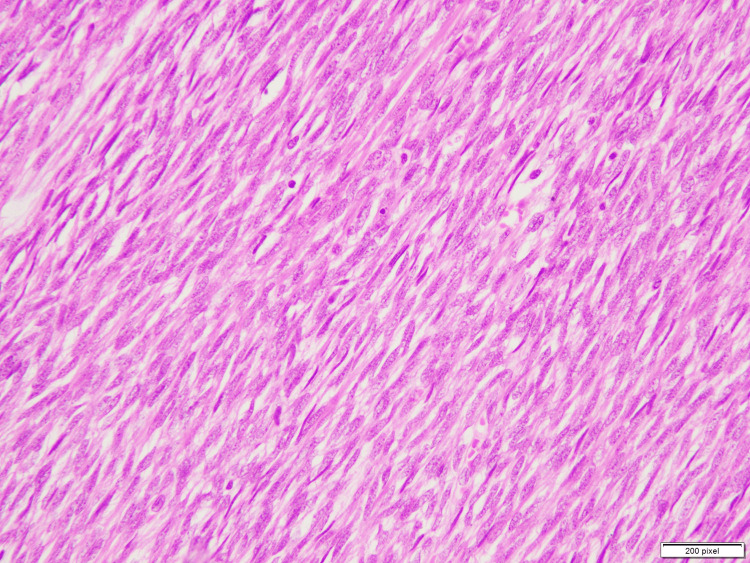
Sections examined reveal a spindle cell lesion showing a hypercellular fascicular architecture with little intervening stroma. Individual cells are monotonous with scant amphophilic cytoplasm, oval to spindle vesicular nuclei and inconspicuous nucleoli.

Sections examined revealed a spindle cell lesion showing a hypercellular fascicular architecture with little intervening stroma. Individual cells are monotonous with scant amphophilic cytoplasm, oval to spindle vesicular nuclei, and inconspicuous nucleoli (Figure 6). Immunohistochemistry was positive for TLE-1 and CD-99, and there was retention of H3K27me3; however, SOX-10 was negative.

Findings favored monophasic synovial sarcoma. The patient was referred back to the surgical clinic for tumor resection and underwent wide local excisions with negative margins. She is doing well in the post-operative period and is scheduled for an oncology appointment for the start of chemotherapy.

## Discussion

Even though synovial sarcomas can occur at all ages, these are more likely to occur in young patients [6] and are commonly seen in the extremities. Chest wall synovial sarcomas is a rare incidence and can be diagnosed by radiography, ultrasound, CT, or MRI. Ultrasound remains the first-line investigation for the evaluation of soft tissue lesions and to characterize the cystic, solid, or mixed solid cystic composition. While smaller lesions of less than 5 cm are homogeneous in echogenicity and are isoechoic to muscles, the larger tumors exhibit a heterogeneous appearance secondary to intralesional cystic/necrotic or hemorrhagic components. Color Doppler shows vascularity within the solid component [7]. Radiography can appear normal in small synovial sarcomas [8], so cross-sectional imaging is more helpful.

CT demonstrates well-circumscribed lesions with intralesional cysts/hemorrhage and a heterogeneous enhancement pattern. Aggressive lesions may invade the chest wall and may be seen as infiltration of the underlying chest wall muscles or destruction of adjacent cortical bone. MRI exhibits heterogeneous signals on T1 and T2 weighted images and heterogeneous post-contrast enhancement. Internal hemorrhage and sedimentary hematoma can result in the appearance of round areas within the fluid-fluid level inside the lesion, forming a bowl-of-fruit appearance [9].

Possible differentials on imaging may include peripheral nerve sheath tumors and solitary fibrous tumors, which cannot be reliably differentiated from synovial sarcoma based on imaging appearances alone; hence, histopathology remains crucial for establishing the exact diagnosis. Synovial sarcomas can show three types of cell appearances: monophasic (consisting of spindle cells), biphasic (comprising spindle and epithelial cells), and poorly differentiated. On the other hand, solitary fibrous tumors demonstrate spindle- or ovoid-shaped cells within a collagenous stroma, intermixed with blood vessels, with a characteristic staghorn shape [10]. Histopathological appearances of peripheral nerve sheath tumors are of low to moderate cellularity, with bland-looking spindled cells containing scanty cytoplasm and oval, elongated, and regular nuclei without nucleoli. These are present randomly in a fibromyxoid stroma containing coarse collagen bundles [11]. 

Treatment is mainly surgical excision followed by chemotherapy and radiotherapy, and because of the high incidence of recurrence, follow-up imaging is crucial. Adjuvant radiotherapy may be considered for larger lesions that surpass 5 cm [1]. Metastasis may be seen in the skin, liver, bone, central nervous system, or breast tissue. Studies have shown better cancer-specific survival for younger patients [1].

## Conclusions

Chest wall synovial sarcomas remain a rare entity, with very few cases described in the literature. Reliable characterization of the lesion can only be made on histopathology, as imaging findings may overlap with other pathologies, such as solitary fibrous tumors and malignant peripheral nerve sheath tumors. Their treatment encompasses a multidisciplinary approach, and radical surgery is associated with a better prognosis. Our case aims to enlighten the audience to keep this differential on the checklist while reviewing the cases of chest wall lesions.
